# Development of Dry and Liquid Duplex Reagent Mix-Based Polymerase Chain Reaction Assays as Novel Tools for the Rapid and Easy Quantification of Bovine Leukemia Virus (BLV) Proviral Loads

**DOI:** 10.3390/v16071016

**Published:** 2024-06-25

**Authors:** Sonoko Watanuki, Kazuyuki Shoji, Masaki Izawa, Mitsuaki Okami, Yingbao Ye, Aronggaowa Bao, Yulin Liu, Etsuko Saitou, Kimikazu Sugiyama, Michiru Endo, Yasunobu Matsumoto, Yoko Aida

**Affiliations:** 1 Laboratory of Global Infectious Diseases Control Science, Graduate School of Agricultural and Life Sciences, The University of Tokyo, 1-1-1 Yayoi, Bunkyo-ku, Tokyo 113-8657, Japan; watanuki-sonoko228@g.ecc.u-tokyo.ac.jp (S.W.);; 2 Molecular Diagnosis Division, Nippon Gene Co., Ltd., 2-8-16 Toiya-machi, Toyama 930-0834, Japan; 3 Hyogo Prefectural Awaji Meat Inspection Center, 49-18 Shitoorinagata, Minamiawaji 656-0152, Japan; 4 Kumagaya Livestock Hygiene Service Center, Kumagaya 360-0813, Japan; 5 Laboratory of Global Animal Resource Science, Graduate School of Agricultural and Life Sciences, The University of Tokyo, 1-1-1 Yayoi, Bunkyo-ku, Tokyo 113-8657, Japan

**Keywords:** bovine leukemia virus (BLV), proviral load (PVL), quantitative real-time PCR (qPCR), duplex, singleplex, liquid, dry

## Abstract

Bovine leukemia virus (BLV) is prevalent worldwide, causing serious problems in the cattle industry. The BLV proviral load (PVL) is a useful index for estimating disease progression and transmission risk. We previously developed a quantitative real-time PCR (qPCR) assay to measure the PVL using the coordination of common motif (CoCoMo) degenerate primers. Here, we constructed a novel duplex BLV-CoCoMo qPCR assay that can amplify two genes simultaneously using a FAM-labeled MGB probe for the BLV LTR gene and a VIC-labeled MGB probe for the *BoLA-DRA* gene. This liquid duplex assay maintained its original sensitivity and reproducibility in field samples. Furthermore, we developed a dry duplex assay composed of PCR reagents necessary for the optimized liquid duplex assay. We observed a strong positive correlation between the PVLs measured using the dry and liquid duplex assays. Validation analyses showed that the sensitivity of the dry duplex assay was slightly lower than that of the other methods for the detection of a BLV molecular clone, but it showed similar sensitivity to the singleplex assay and slightly higher sensitivity than the liquid duplex assay for the PVL quantification of 82 field samples. Thus, our liquid and dry duplex assays are useful for measuring the BLV PVL in field samples, similar to the original singleplex assay.

## 1. Introduction

Bovine leukemia virus (BLV) is the causative agent of enzootic bovine leukemia (EBL), the most common neoplastic disease in cattle. It belongs to the genus *Deltaretrovirus* of the family *Retroviridae* and is closely related to human T-cell leukemia virus types 1 and 2 [1]. Similar to other retroviruses, BLV integrates into the host genome as a provirus and causes lifelong infections. Approximately 70% of BLV-infected cattle show no clinical symptoms, whereas 30% of infected cattle develop persistent lymphocytosis and 2–5% of BLV-infected cattle form B-cell lymphoma after a long latency period [1]. BLV is highly prevalent in most regions, except for some European countries and Oceania [2]. For example, the proportion of BLV-positive cattle ranges from 28.0 to 40.9% in Japan [3], 40.0 to 48.0% in Korea [4], 31.0 to 41.9% in China [5,6], 11.0 to 100% in Thailand [7], 4.8 to 9.7% in the Philippines [8], 21.5 to 28.0% in Egypt [9,10], 37.04% in Myanmar [11], more than 40.0% in the USA [12], and 88.4% of dairy herds in Canada [13]. BLV infects cattle worldwide and has a severe economic impact on the cattle industry [14,15,16]. However, no effective treatments or commercially available vaccines exist [17]. Therefore, segregating or culling infected animals from herds and preventing herds from becoming newly infected are considered the most effective prevention or eradication strategies against BLV [18].

In general, BLV infection in cattle is identified using two types of BLV diagnostic methods: (1) serological methods such as enzyme-linked immunosorbent assay (ELISA) and (2) BLV proviral DNA detection methods using PCR [19]. ELISA is widely used to detect anti-BLV antibodies because of its high sensitivity and relatively easy operation. However, it has the following problems: (1) neonatal calves cannot undergo antibody testing because they have transitional antibodies for six months after birth; (2) BLV-infected cattle with low, transient, or absent antibody titers cannot be diagnosed using serological tests; and (3) information regarding the degree of BLV-induced disease progression cannot be provided [20]. In contrast, the BLV provirus remains integrated into the host genome with or without antibodies [21] and can be amplified after a period of latency [21,22]. Therefore, in addition to serological methods, diagnostic BLV PCR techniques that detect the BLV proviral genome integrated within the host genome are used to detect BLV infection. In particular, many studies have shown that BLV proviral loads (PVLs), which are the number of copies of a provirus, are associated with disease progression [23,24,25] and transmission risk [23,26,27,28,29], suggesting that determination of the PVL is important as a useful diagnostic marker. It has been reported that BLV-infected cows with high PVLs are at risk of spreading the virus [30,31,32,33] and EBL development [23,34,35]. Previously, we developed a highly specific, accurate, and sensitive qPCR method to quantify BLV PVLs for both known and novel BLV variants using the coordination of common motifs (CoCoMo) degenerate primers [20,23]. This assay is a TaqMan probe-based real-time PCR method targeting the BLV LTR region, which is present in two copies per provirus and contributes to the improved sensitivity of our assay. Furthermore, it has the advantage of normalizing viral genomic DNA by amplifying it in parallel with the bovine leukocyte antigen (*BoLA*)-*DRA* gene as a single-copy host gene to express the BLV PVLs as the number of copies per cell. Thus, this assay can allow for adjustment of variations in the amplification efficiency between samples and comparison of the BLV PVLs measured at different institutions and using different PCR instruments [20,23]. Moreover, we recently developed the BLV-CoCoMo-qPCR-2 to improve the original BLV-CoCoMo-qPCR by optimizing the primer degeneracy and PCR conditions and reconstructing a standard plasmid [36]. This BLV-CoCoMo-qPCR-2 assay is currently used to carry out the integrated BLV eradication strategy, contributing greatly to BLV infection control. Interestingly, previous studies have shown that the BLV-CoCoMo-qPCR-2 assay can detect BLV proviruses in the milk, nasal mucus, and saliva of dairy cattle with PVLs of >10,000, 14,000, and 18,000 copies/10^5^ cells in matched blood, respectively [31,32]. Therefore, technologies to quantify BLV PVLs are crucial for estimating the BLV transmission risk and controlling and eradicating BLV infections.

Constructing a simple and user-friendly dual-target detection system that can detect target genes and internal controls simultaneously in the same reaction has become highly desirable. Screening a large population using a singleplex qPCR assay is laborious and resource-intensive; thus, a duplex qPCR assay is required to detect the target and host genes simultaneously in the same reaction, which saves time and money [37]. Furthermore, a duplex qPCR assay can avoid human error by reducing the number of pipetting steps required. The first objective of the present study was to establish a novel BLV-CoCoMo Dual qPCR (Liquid Dual-CoCoMo assay) that allows the simultaneous detection of two genes, the BLV LTR region and the *BoLA-DRA* gene, in the same reaction. The second objective was to develop a dried BLV-CoCoMo Dry Dual qPCR reagent (Dry Dual-CoCoMo assay) containing the reagents required for the Dual-CoCoMo assay to simplify the handling of the PCR preparation and facilitate the diagnosis of BLV. Finally, we validated the accuracy of the novel Liquid Dual-CoCoMo assay and the Dry Dual-CoCoMo assay by determining their limits of detection and comparing them with the original BLV-CoCoMo-qPCR-2 assay (Single-CoCoMo assay), and then we evaluated whether these two novel assays are clinically applicable to field samples. The results of the present study using our newly developed Liquid and Dry Dual-CoCoMo assays would enable us to reduce the labor time, costs, and human pipetting errors, as well as to eliminate the reagent storage and transportation constraints for detecting BLV infections and quantifying the BLV PVL.

## 2. Materials and Methods

### 2.1. Animal Samples, DNA Extraction, and Plasma Isolation

Blood samples were collected from 82 dairy and beef cattle on BLV-positive farms in Japan, including two BLV-infected cattle with lymphoma. Genomic DNA was extracted from ethylenediaminetetraacetic acid (EDTA)-treated whole blood samples using a Wizard Genomic DNA Purification Kit (Promega Corporation, Tokyo, Japan) according to the manufacturer’s instructions. The concentrations of the genomic DNA samples were adjusted to 30 ng/µL for the qPCR assays according to the protocol of the Single-CoCoMo assay. Portions of the EDTA-treated whole blood samples were used to separate plasma for the detection of anti-BLV antibodies. All the animals were handled by veterinarians in accordance with the University of Tokyo’s guidelines. This study was approved by the Animal Experiments Committee of the University of Tokyo (Approval Number p22–2–030).

### 2.2. Detection of Anti-BLV Antibodies in Plasma Samples

An anti-BLV antibody ELISA kit (Nippon Gene Co., Ltd., Toyama, Japan) was used to detect anti-Env gp51 antibodies according to the manufacturer’s instructions.

### 2.3. Determination of the BLV PVL Using Single-CoCoMo Assay

The BLV PVLs were determined using a BLV-CoCoMo-qPCR-2 assay (Nippon Gene) using a THUNDERBIRD Probe qPCR Mix (Toyobo, Tokyo, Japan), as described previously [36]. Briefly, the BLV LTR gene was amplified using the degenerate CoCoMo primer mix for the BLV LTR gene and detected using a 6-carboxyfluorescein (FAM)-labeled minor groove binder (MGB) probe. As an internal control, the *BoLA-DRA* region was amplified using a primer mix for the *BoLA-DRA* gene and detected using a FAM-labeled MGB probe. All the amplification steps were performed on a LightCycler^®^ 480 System II (Roche Diagnostics, Mannheim, Germany). Finally, the PVL was calculated using the following formula: (number of BLV LTR copies/number of *BoLA-DRA* copies) × 10^5^ cells. The BLV PVL was expressed as the number of copies per 10^5^ cells.

### 2.4. Development and Optimization of the Liquid Dual-CoCoMo Assay

We developed and optimized a Liquid Dual-CoCoMo assay based on the original BLV-CoCoMo-qPCR-2 method. First, we prepared two MGB probes labeled with different fluorescent dyes (FAM and VIC) to detect two target genes in the same reaction. We used previously reported sequences of the MGB probes [36] (Supplementary Table S1). Briefly, the sequences of the FAM-LTR and VIC-DRA probes were 5′-FAM-CTCAGCTCTCGGTCC-NFQ-MGB-3′ (BLV LTR gene) and 5′-VIC-TGTGTGCCCTGGGC-NFQ-MGB-3′ (*BoLA-DRA* gene), respectively. The primers used for the Dual-CoCoMo assay were previously reported as degenerate CoCoMo primers (Nippon Gene) (Supplementary Table S1).

We used the following as the qPCR reagent for the Liquid Dual-CoCoMo assay: (1) THUNDERBIRD^®^ Probe qPCR Mix (TOYOBO), which was used for the Single-CoCoMo assay, and (2) GeneAce Probe qPCR Mix II (Nippon Gene). The PCR was performed in 20 µL reactions containing 1.25 µL of the CoCoMo-BLV primer mix and CoCoMo-DRA primer mix (Nippon Gene), 1.25 µL of the TaqMan MGB probe for FAM-LTR and VIC-DRA, 10 µL of a 2×qPCR master mix (provided by each company) and 5 µL of genomic DNA adjusted to 30 ng/µL. The PCR conditions for TBIRD were initially 1 min at 95 °C, followed by 45 cycles at 95 °C for 15 s and 60 °C for 1 min. The PCR conditions for GeneAce were 10 min at 95 °C, followed by 45 cycles at 95 °C for 15 s and 60 °C for 1 min. We assessed the intra- and inter-assay variations to compare the precision of the two qPCR master mixes for the Liquid Dual-CoCoMo assay. We chose genomic DNA samples from ten cows with different PVLs from the field samples. Each sample was measured in triplicate, and the PCRs were repeated three times. The coefficient of variation (CV) was calculated as an indicator of variability by using the determined PVLs. CV values < 20% were defined as acceptable in this study based on the assay CV range of our previous study [23].

### 2.5. Development of the Dry Dual-CoCoMo Assay

All the qPCR components were improved based on the GeneAce qPCR master mix and freeze-dried in 0.2 mL PCR tubes (Nippon Gene). Lyophilization of the qPCR master mix was performed at Nippon Gene Co., Ltd. The dried reagents were stored at room temperature until use. Quantitative PCR was performed by adding 15 µL of PCR-grade water and 5 µL of genomic DNA adjusted to 30 ng/µL to the 0.2 mL tubes containing the dried reagents. The mixture was vortexed and centrifuged briefly to dissolve the dried pellet.

### 2.6. Comparison of the Sensitivity of the Liquid Dual-CoCoMo Assay and Dry Dual-CoCoMo Assay

The analytical sensitivities of the Liquid and Dry Dual-CoCoMo assays were validated by comparison with that of the Single-CoCoMo assay using an infectious full-length molecular clone of BLV (pBLV-IF2) [38,39]. Briefly, pBLV-IF2 was serially diluted two-fold with Tris-EDTA (TE) buffer to obtain a range of provirus copy numbers from 100 to 0.78125. Each diluted sample was supplemented with 150 ng of genomic DNA from a BLV-uninfected cow. The analytical sensitivity of the three methods was examined after triplicate PCR amplifications as the percentage of successful amplifications.

### 2.7. Validation of Assay Accuracy for the Clinical Applicability Using Field Samples

We determined the diagnostic specificities and sensitivities of the Liquid and Dry Dual-CoCoMo assays using 82 genomic DNA samples from cows in the field as a comparison with the results of the Single-CoCoMo assay. The correlation between the PVLs measured by these two novel assays and the Single-CoCoMo assay was validated using BLV-infected cows.

### 2.8. Statistical Analysis

Dunnett’s multiple comparison test was used to compare the mean BLV PVL for all the combinations of the Single-CoCoMo assay (control) and Liquid Dual-CoCoMo assays using the TBIRD and GeneAce groups. Pearson’s correlation was used to assess the strength of the association of the quantitative values between the two assays. All the analyses were performed using the R software (version 4.3.2).

## 3. Results

### 3.1. Construction and Optimization of the Liquid Dual-CoCoMo Assay

**Figure 1:**
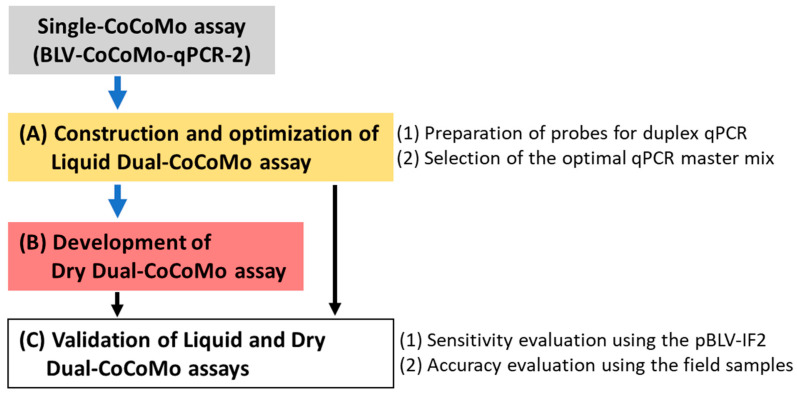
Flowchart of the experimental procedure in this study. (**A**) Based on the BLV-CoCoMo-qPCR-2 (Single-CoCoMo) assay, the first step (yellow box) was to construct an MGB probe labeled with FAM dye to detect the BLV LTR gene and an MGB probe labeled with VIC dye to detect the *BoLA-DRA* gene. This was performed to simultaneously detect the two genes in the same reaction well and to select the optimal qPCR master mix for the novel BLV-CoCoMo Dual qPCR (Liquid Dual-CoCoMo assay) reported in this study. (**B**) The second step (pink box) was to develop a ready-to-use Dry Dual-CoCoMo assay for simplifying the PCR preparation process. (**C**) The third step was to evaluate the sensitivity of the Liquid Dual-CoCoMo and Dry Dual-CoCoMo assays using the pBLV-IF2 and field samples by comparing the results with that of the Single-CoCoMo assay. In addition, correlation analysis was also conducted to confirm the relationship between the proviral loads measured by the novel two assays and that measured by the Single-CoCoMo assay.

Our previously developed BLV-CoCoMo-qPCR-2 assay has been widely used by BLV researchers and veterinarians [24,25] due to its quantitative nature and high specificity, sensitivity, and reproducibility. However, because that assay uses only MGB probes, which are labeled with FAM dye for detecting the BLV LTR and *BoLA-DRA* genes [36], these two target genes cannot be detected simultaneously in the same qPCR reaction well using this assay. To overcome this limitation, we developed the Liquid Dual-CoCoMo assay in this study (Figure 1A).

**Figure 2:**
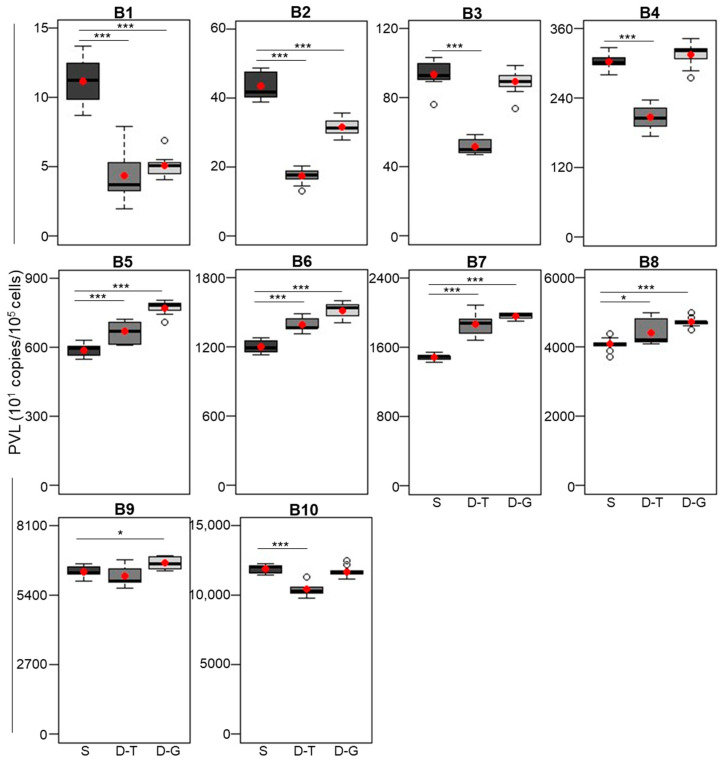
Comparison of the BLV proviral load (PVL) measured by the BLV-CoCoMo Dual qPCR (Liquid Dual-CoCoMo assay) using two qPCR master mixes and that of the BLV-CoCoMo-qPCR-2 (Single-CoCoMo assay). The PVLs of ten BLV-infected cattle (B1–B10) were amplified in triplicate from those samples in three independent experiments using the Liquid Dual-CoCoMo assay with a THUNDERBIRD Probe qPCR master mix (TBIRD) and GeneAce Probe qPCR mix II (GeneAce) (*n* = 9). Box plots of S (dark gray), D-T (gray), and D-G (light gray) represent the PVLs measured by the Single-CoCoMo assay, the Liquid Dual-CoCoMo assay with TBIRD, and the Liquid Dual-CoCoMo assay with GeneAce, respectively. The upper bar indicates the maximum value and the lower bar shows the minimum values. The means are denoted by the red circles and the medians are shown as black bars. The *p*-values were calculated using Dunnett’s test. Asterisks indicate significant differences (* *p* < 0.05 and *** *p* < 0.001).

We constructed an MGB probe labeled with FAM to detect the BLV LTR gene and an MGB probe labeled with VIC to detect the *BoLA-DRA* gene (Figure 1A-(1)). To select the optimal qPCR master mix for the Liquid Dual-CoCoMo assay, we tested the following two commercial reagents: THUNDERBIRD Probe qPCR Mix (TBIRD) and GeneAce Probe qPCR Mix II (GeneAce) (Figure 1A-(2)). Using the Single-CoCoMo assay for comparison with the Liquid Dual-CoCoMo assay, we measured the PVLs in triplicate using the genomic DNA of ten BLV-infected cattle and repeated the assay three times (Table 1). The PVLs were successfully determined using the Liquid Dual-CoCoMo assay with two qPCR master mixes: PVLs ranging from 44 to 104,926 copies per 10^5^ cells for TBIRD, and from 51 to 116,959 copies per 10^5^ cells for GeneAce (*n* = 10 animals). Similarly, the PVLs ranged from 112 to 119,317 copies per 10^5^ cells in the Single-CoCoMo assay. However, in samples B1, B2, B3, and B4, which showed less than 3000 copies per 10^5^ cells, the PVLs measured by the Liquid Dual-CoCoMo assay using TBIRD were significantly lower than those of the Single-CoCoMo assay in all four samples. In contrast, the PVLs measured by the Liquid Dual-CoCoMo assay with GeneAce were significantly lower than those of the Single-CoCoMo assay in only samples B1 and B2 (Figure 2). In addition, the PVL of sample B10, as measured by the Liquid Dual-CoCoMo assay using TBIRD, was lower than that measured by a Single-CoCoMo assay. In contrast, the PVLs of samples B5, B6, B7, B8, and B9, as measured by the Liquid Dual-CoCoMo assay using GeneAce, were significantly higher than those of the Single-CoCoMo assay, whereas those of samples B3, B4, and B10 showed no difference in quantitative values between these methods. In addition, the PVLs of samples B5, B6, B7, and B8 measured by the Liquid Dual-CoCoMo assay using TBIRD were significantly higher than those measured by the Single-CoCoMo assay, whereas the B9 samples showed no difference in quantitative values between these methods.

**Figure 3:**
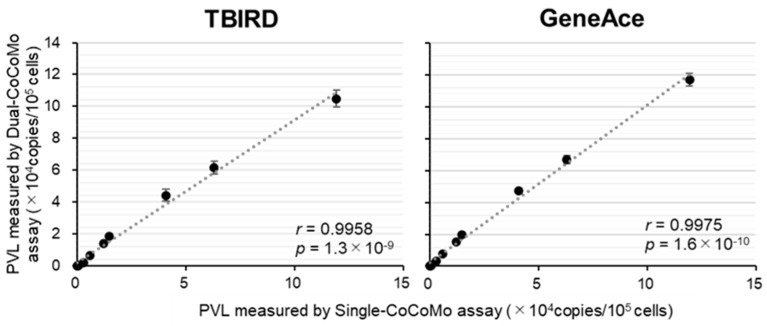
Correlation between the BLV proviral load (PVL) measured by the BLV-CoCoMo Dual qPCR using two different qPCR master mixes and that of BLV-CoCoMo-qPCR-2. The PVLs of ten BLV-infected cattle (B1–B10) were amplified from the corresponding samples in three independent experiments using the Liquid Dual-CoCoMo assay with a THUNDERBIRD Probe qPCR master mix (TBIRD) or a GeneAce Probe qPCR Mix II (GeneAce). The correlation between the mean PVLs measured by the Liquid Dual-CoCoMo assay using TBIRD or GeneAce and that by the Single-CoCoMo assay was evaluated using Pearson’s correlation coefficient (*r*). The *p*-values are indicated in the graphs. The dotted line represents the approximate curve.

Furthermore, we examined the correlation between the quantitative values of the PVLs measured by the Liquid Dual-CoCoMo assay using TBIRD or GeneAce and those measured by the Single-CoCoMo assay. As shown in Figure 3, these results reveal a strong positive correlation, with correlation coefficients (*r*) of 0.9958 and 0.9975, respectively. This result indicates that the PVLs measured by the Liquid Dual-CoCoMo assay using GeneAce were more highly correlated with the PVLs in the Single-CoCoMo assay than measured with the Liquid Dual-CoCoMo assay using TBIRD.

To verify the reproducibility of the Liquid Dual-CoCoMo assay using the two qPCR master mixes, we assessed their intra- and inter-assay variations (Table 2). We performed PCR amplifications in triplicate for each sample, and the assay was repeated three times using genomic DNA from ten BLV-infected cattle. The intra-assay CV for PVLs in the Single-CoCoMo assay ranged from 0.9% to 16.9%; the CV of the Liquid Dual-CoCoMo assay using TBIRD and GeneAce ranged from 0.4% to 29.9% and 0.6% to 18.1%, respectively. The inter-assay CV for PVLs in the Single-CoCoMo assay ranged from 0.9% to 16.1%, whereas the CV of the Liquid Dual-CoCoMo assay using TBIRD and GeneAce ranged from 1.4% to 48.4% and 1.2% to 10.4%, respectively. This result indicates that the Liquid Dual-CoCoMo assay using TBIRD showed large variations for the B1 sample with low PVLs in the intra- and inter-assays. These results demonstrate that the Liquid Dual-CoCoMo assay with GeneAce has good intra- and inter-assay reproducibility, similar to that of the Single-CoCoMo assay.

Taken together, we chose the GeneAce qPCR master mix for the Liquid Dual-CoCoMo assay in subsequent experiments.

### 3.2. Development of the Dry Dual-CoCoMo Assay and Correlation Analysis with the Liquid Dual-CoCoMo Assay

**Figure 4:**
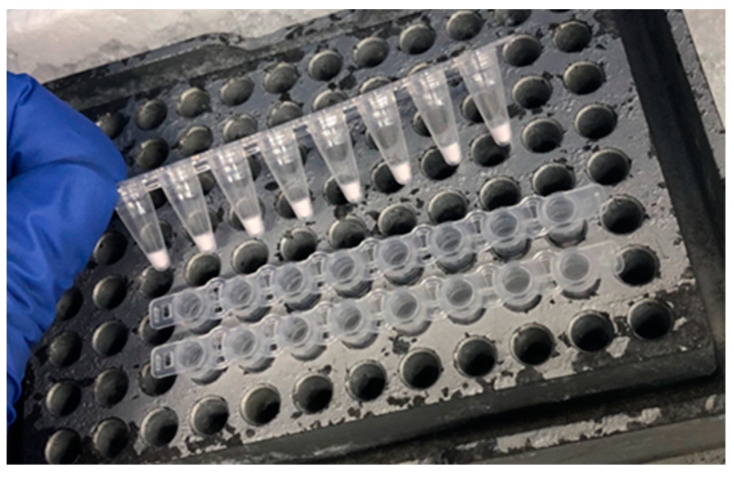
Appearance of the ready-to-use BLV-CoCoMo Dry Dual qPCR reagent. All the PCR reagents needed for the Liquid Dual-CoCoMo assay containing the CoCoMo primer mix and TaqMan MGB probes for the BLV LTR gene and the *BoLA-DRA* gene, and the qPCR master mix were dried in one PCR reaction tube. This reagent was produced by Nippon Gene Co., Ltd. (Toyama, Japan).

**Figure 5:**
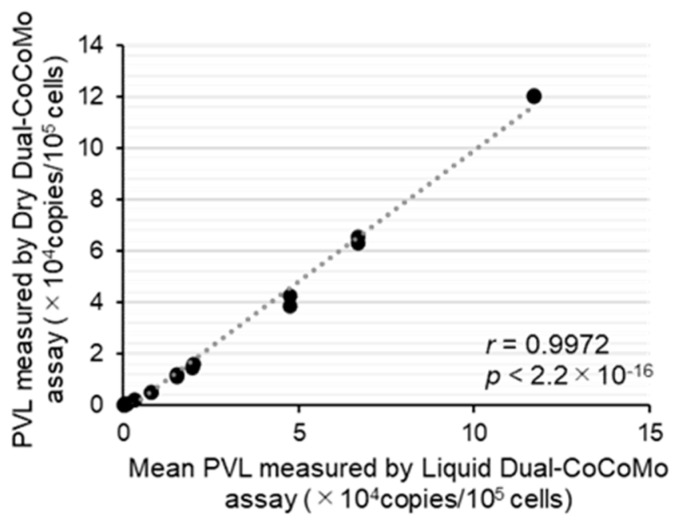
Correlation between the BLV proviral load (PVL) measured by the Dry Dual-CoCoMo assay and the mean PVL measured by the Liquid Dual-CoCoMo assay. The PVL of ten BLV-infected cattle (B1–B10) were amplified in duplicate using the Dry Dual-CoCoMo assay. The correlation between the PVLs measured by the Dry Dual-CoCoMo assay and the mean PVLs measured by the Liquid Dual-CoCoMo assay was evaluated using Pearson’s correlation coefficient (*r*). The *p*-values are indicated in the graphs. The dotted line represents the approximate curve.

To facilitate and expedite the PCR testing workflow with stable accuracy and precision, we developed a ready-to-use BLV-CoCoMo Dry Dual qPCR reagent using the optimized Liquid Dual-CoCoMo assay (Figure 1B). The dry reagents included improved PCR components based on the GeneAce qPCR master mix, which are necessary for the Dual-CoCoMo assay (Figure 4). To compare the accuracy of the Dry Dual-CoCoMo assay and the Liquid Dual-CoCoMo assay, we performed PCR amplifications in duplicate of the genomic DNA of 10 BLV-infected cattle using the Dry Dual-CoCoMo assay. The BLV PVLs of all ten BLV-infected cows were successfully determined using the Dry Dual-CoCoMo assay, with values ranging from 15 to 120,377 copies per 10^5^ cells (Figure 5). Next, assuming that the average PVLs determined by the Liquid Dual-CoCoMo assay with GeneAce (Table 1) were the true values, we evaluated the correlation between the quantitative values of the PVLs determined by the Dry Dual-CoCoMo assay and the average PVLs determined by the Liquid Dual-CoCoMo assay. As shown in Figure 5, a strong positive correlation was observed for the PVLs measured by the two Dual-CoCoMo assays (correlation coefficient (*r*) = 0.9972 [*p* = 2.2 ×10^−16^]), indicating that the PVLs measured by the Dry Dual-CoCoMo assay and the Liquid Dual-CoCoMo assay were concordant.

### 3.3. Validation of the Accuracy of the Liquid Dual-CoCoMo Assay and Dry Dual-CoCoMo Assay on BLV Provirus Detection

To elucidate the analytical sensitivity of the Dry and Liquid Dual-CoCoMo assays, we compared them with that of the Single-CoCoMo assay by detecting BLV provirus using an infected full-length molecular clone of BLV (pBLV-IF2) (Figure 1C-(1)). We performed a two-fold dilution of pBLV-IF2, adjusted the proviral copy number from 100 to 0.78125 per 10^5^ cells, performed three or ten PCR amplifications, and verified the BLV detection percentage. As shown in Table 3, when present at 12.5 copies or more, all the methods showed the same detection rate and detected 100% (3/3) of pBLV-IF2. In contrast, when present at 6.25 copies, the Single-CoCoMo assay and the Liquid Dual-CoCoMo assay detected 100% (3/3) of the pBLV-IF2; however, the detection rate was lower with the Dry Dual-CoCoMo assay, at 67% (2/3). When present at 3 to 0.78125 copies, the Liquid Dual-CoCoMo assay had a slightly lower detection rate than the Single-CoCoMo assay but could detect 10% of 0.78125 copies of pBLV-IF2. In contrast, the Dry Dual-CoCoMo assay had a detection rate of 70% (7/10) for pBLV-IF2 when present at 3.125 copies and a rate of 30% (3/10) when present at 1.5625 copies; however, 0% (0/10) of pBLV-IF2 was detected when present at 0.78125 copies.

### 3.4. Validation of Accuracy Liquid Dual-CoCoMo Assay and Dry Dual-CoCoMo Assay on Field Samples

To evaluate the accuracy of the Liquid and Dry Dual-CoCoMo assays, we compared the results with those of the Single-CoCoMo assay by determining the BLV PVL using field samples (Figure 1C-(2)). We also tested all the samples with BLV-ELISA to detect BLV infection. A total of 70 out of the 82 cows tested positive for BLV antibodies (Table 4). In parallel, BLV provirus was detected in 71, 70, and 71 of the 82 cows using the Single-CoCoMo assay, Liquid Dual-CoCoMo assay, and Dry Dual-CoCoMo assay, respectively (Table 5). Eleven cows tested negative in both the ELISA and PCR. One cow (S40) was ELISA-negative and PCR-positive (Table 4). In addition, none of the cows tested ELISA-positive and PCR-negative (Table 4). Next, assuming that the Single-CoCoMo assay is the criterion standard, we compared the diagnostic specificities and sensitivities of the Liquid and Dry Dual-CoCoMo assays for the field samples (Table 5). The results showed that 70 cattle (sensitivity: 98.6%; 95% CI: 92.4–100%) and 71 cattle (sensitivity: 100%; 95% CI: 94.9–100%) were positive among 71 samples using the Liquid and Dry Dual-CoCoMo assays, respectively (Table 5). This result indicates that the Dry Dual-CoCoMo assay detected the BLV provirus in field samples with higher sensitivity than the Liquid Dual-CoCoMo assay.

**Figure 6:**
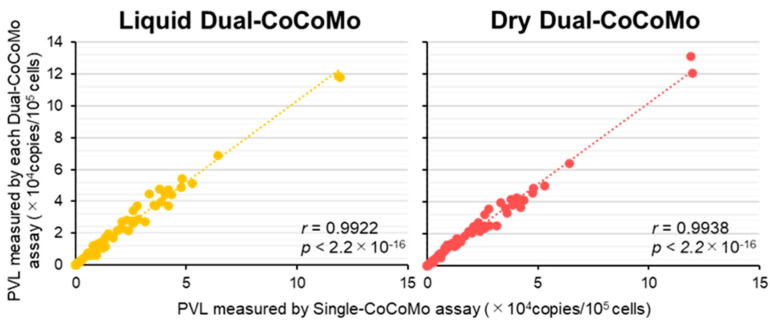
Correlation between the BLV proviral loads (PVLs) in BLV-infected cows as measured using the two novel Dual-CoCoMo assays and the Single-CoCoMo assay. The PVLs of 71 BLV-infected cows, including 2 BLV-infected cows with lymphoma, were determined in duplicate using the Liquid Dual-CoCoMo assay and the Dry Dual-CoCoMo assay. The correlation between the mean PVLs measured by the Liquid Dual-CoCoMo assay or the Dry Dual-CoCoMo assay and that measured by the Single-CoCoMo assay was evaluated using Pearson’s correlation coefficient (*r*). The *p*-values are indicated in the graphs. The dotted line represents the approximate curve.

Furthermore, using samples from 71 BLV-infected cows, including 2 BLV-infected cows with lymphoma, we examined the correlation between the quantitative values of the PVLs measured by the Liquid Dual-CoCoMo assay or Dry Dual-CoCoMo assay and those measured by the Single-CoCoMo assay (Figure 6). The result showed that the correlation coefficients were 0.9922 and 0.9938 for the Liquid and Dry Dual-CoCoMo assays, respectively, indicating a strong correlation between the two methods. These results clearly suggest that the Liquid Dual-CoCoMo and Dry Dual-CoCoMo assays are useful for the measurement of the BLV PVL, as well as the Single-CoCoMo assay.

### 3.5. Comparison of the Characteristic Features of the Single-CoCoMo, Liquid Dual-CoCoMo and Dry Dual-CoCoMo Assays

**Figure 7:**
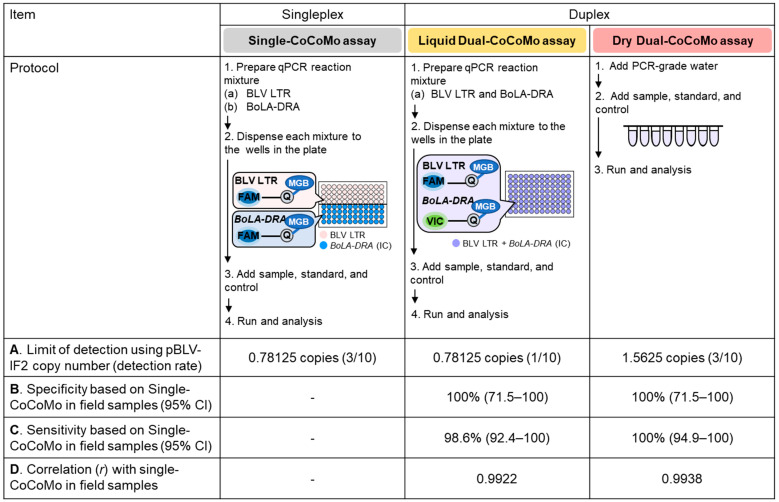
Summary of the characteristics of the Single-CoCoMo, Dual-CoCoMo, and Dry Dual CoCoMo assays. Protocols for the Single-CoCoMo, Liquid Dual-CoCoMo, and Dry Dual-CoCoMo assays and values for the following parameters are presented. (**A**) Limits of detection (detection rate) for the three assays using pBLV-IF2. (**B**) Assay diagnostic specificity (95% CI) using field samples based on the Single-CoCoMo assay. (**C**) Assay diagnostic sensitivity (95% CI) using field samples based on the Single-CoCoMo assay. (**D**) Correlation between the BLV proviral loads of 71 BLV-infected cows as detected using the two novel Dual-CoCoMo assays and the Single-CoCoMo assay. The Pearson correlation coefficient (*r*) is shown.

A comparison of the characteristic features of the three CoCoMo assays investigated in this study is summarized in Figure 7. The two novel Dual-CoCoMo assays and the original Single-CoCoMo assay strongly correlated and had the same specificity for measuring the BLV PVL in field samples. However, the analytical sensitivity of the Dry Dual-CoCoMo assay was slightly lower than those of the other assays for detecting the BLV proviral clone pBLV-IF2. Furthermore, it showed the same diagnostic sensitivity as the Single-CoCoMo assay and was slightly higher than the Liquid Dual-CoCoMo assay for quantifying the BLV PVL in field samples. Moreover, the diagnostic sensitivity of the Liquid Dual-CoCoMo assay was slightly lower than that of the other assays for quantifying samples with low PVLs, which were near the limit of detection of the BLV provirus. As described here, the two novel Dual-CoCoMo assays appear to have similar specificities and sensitivities to the original Single-CoCoMo assay.

## 4. Discussion

Four major conclusions can be drawn from the improvement to the BLV-CoCoMo-qPCR-2 assay in this study. First, based on the original Single-CoCoMo assay, we successfully constructed a novel Liquid Dual-CoCoMo assay that allowed us to amplify two genes simultaneously in the same well using a FAM-labeled MGB probe for the BLV LTR gene and a VIC-labeled MGB probe for the *BoLA-DRA* gene. We also selected the GeneAce Probe qPCR mix II, but not the THUNDERBIRD Probe qPCR mix, which is usually used for the Single-CoCoMo assay. Interestingly, our newly developed Liquid Dual-CoCoMo assay clearly showed a high correlation between the PVLs of 10 BLV-infected cows measured using the Single-CoCoMo assay and good intra- and inter-assay reproducibility, like the Single-CoCoMo assay. Although the Single-CoCoMo assay can accurately measure the BLV PVL by amplifying the *BoLA-DRA* gene in parallel with the BLV LTR gene using each FAM-labeled MGB probe, it is laborious and resource-intensive. Therefore, improving the BLV-CoCoMo-qPCR-2 assay will reduce the labor time, costs, and human pipetting errors, making the BLV provirus detection system more widely available. Second, we developed the Dry Dual-CoCoMo assay, which is composed of PCR reagents containing the GeneAce qPCR master mix necessary for the optimized Liquid Dual-CoCoMo assay. We observed a strong positive correlation between the PVLs measured by the Liquid Dual-CoCoMo assay and this Dry Dual-CoCoMo assay. To the best of our knowledge, this is the first report on the use of dried reagents in a quantitative method for BLV, which allows the elimination of the reagent storage and transportation constraints. Third, validation analysis of the accuracy of the two novel assays demonstrated a strong correlation between both methods and the Single-CoCoMo assay, and the same specificity as the Single-CoCoMo assay. However, the Dry Dual-CoCoMo assay has a slightly lower analytical sensitivity than the Liquid Dual-CoCoMo assay and the Single-CoCoMo assay for detecting the BLV proviral clone pBLV-IF2 despite it having a slightly higher diagnostic sensitivity for quantifying field samples. Finally, our results demonstrated that the two novel Dual-CoCoMo assays have similar diagnostic specificity and sensitivity to the original Single-CoCoMo assay and a strong positive correlation between the PVLs measured by the two novel Dual-CoCoMo assays and the Single-CoCoMo assay. Therefore, our newly developed Liquid Dual-CoCoMo and Dry Dual-CoCoMo assays will significantly contribute to the control and eradication of BLV.

Initially, we established a novel Liquid Dual-CoCoMo system with a simple and user-friendly design that allowed the simultaneous detection of two genes in the same reaction well. It has been reported that the DNA polymerase and buffer within a qPCR master mix affect the amplification efficiency and the ability to detect specific DNA sequences [40]. Thus, we compared two commercial qPCR master mixes, the TBIRD qPCR master mix and the GeneAce qPCR master mix, for the Dual-CoCoMo assay. The TBIRD qPCR master mix, which has high sensitivity, specificity, and cost-effectiveness, is usually used for the Single-CoCoMo assay [10,11,29,31,33,34,41,42,43,44,45]. In addition, the TBIRD qPCR master mix has been used in several multiplex qPCR assays [46]. Contrary to our expectations, in this study, the use of TBIRD in the Liquid Dual-CoCoMo assay showed reduced measurement accuracy compared with the GeneAce-based Dual CoCoMo assay. For example, the PVLs measured by the Liquid Dual-CoCoMo assay using TBIRD were lower than those measured by GeneAce in all 10 tested samples (Table 1). In addition, comparing the inter-assay coefficients of variability for the PVLs, the Liquid Dual-CoCoMo assay using TBIRD showed a high CV value and less stable values than GeneAce (Table 2). Although the reasons for these results are unclear, the THUNDERBIRD Next Probe qPCR Mix (TOYOBO) optimized for multiplex qPCR has been released, and its use may have improved the results of our Liquid Dual-CoCoMo assay (https://lifescience.toyobo.co.jp/detail/detail.php?product_detail_id=276; accessed on 7 May 2024). Meanwhile, the GeneAce qPCR master mix, which showed high accuracy and reproducibility in the Liquid Dual-CoCoMo assay, can be used in multiplex qPCR such as single nucleotide polymorphism (SNP) genotyping assays. It is characterized by high specificity and amplification efficiency, and by low cost. Indeed, when the GeneAce qPCR master mix was used for the Liquid Dual-CoCoMo assay, stable quantification was achieved: the quantitative values were similar to those of the Single-CoCoMo assay and the assay reproducibility was equal to or better than that of the Single-CoCoMo method, indicating that GeneAce is a good choice for the Liquid Dual-CoCoMo assay. In particular, the CV value of the Liquid Dual-CoCoMo assay with GeneAce for detecting low-copy specimens was lower than that of the Single-CoCoMo assay, suggesting that using a duplex-PCR technique reduced the assay variability in detecting low-copy specimens.

For the first time, we succeeded in expanding the original BLV-CoCoMo-qPCR-2 assay from a Liquid Dual-CoCoMo assay to a Dry Dual-CoCoMo assay to reduce the workload further and improve the efficiency. A summary of the characteristic features of the Single-CoCoMo, Liquid Dual-CoCoMo, and Dry Dual-CoCoMo assays is provided in Figure 7, which shows that the two Dual-CoCoMo assays retained the similar specificity and sensitivity to the Single-CoCoMo assay. The PVLs measured between the two Dual-CoCoMo assays and the Single-CoCoMo assay showed a strong correlation. Our results clearly demonstrate that these methods can be used in a manner similar to the original method. The high sensitivity observed can be attributed to the use of degenerate primers that target all the available sequences within a specific region (BLV LTR) characterized by the CoCoMo method [23]. In detail, the Liquid Dual-CoCoMo assay was estimated to have comparable analytical sensitivity to the original assay, since up to 0.78125 copies of pBLV-IF2 were detected, similar to the Single-CoCoMo assay. However, the detection rate for the pBLV-IF2 with 0.78125 copies was slightly lower (1/10; 10%) using the Liquid Dual-CoCoMo assay than the Single-CoCoMo assay (3/10; 30%). In addition, the Dry Dual-CoCoMo assay could only detect up to 1.5625 copies of pBLV-IF2, which was less sensitive than the Single-CoCoMo and Liquid Dual-CoCoMo assays. It is possible that using a multiplex qPCR assay may have resulted in reduced sensitivity compared to singleplex assays due to the self-inhibition among different sets of primers [47]. Interestingly, the Dry Dual-CoCoMo assay can also be considered a qPCR assay with high analytical sensitivity for BLV provirus detection because it has been reported that a general qPCR assay using the TaqMan assay for BLV diagnosis detects at least 1–10 copies of the nucleic acid template (BLV plasmid) per reaction [48,49]. Regarding their diagnostic sensitivity to field samples, the Dry Dual-CoCoMo assay had the same detection rate as the Single-CoCoMo assay. However, the Liquid Dual-CoCoMo assay could not detect a sample with a low PVL, S23, among all the positive samples detected by the Single-CoCoMo and Dry Dual-CoCoMo assays. Thus, the Dry Dual-CoCoMo assay was considered to have a higher diagnostic sensitivity than the Liquid Dual-CoCoMo assay for detecting field samples despite its rather low analytical sensitivity for detecting the pBLV-IF2 provirus.

Our results using 82 field samples showed that 1 cow (S23) out of the 71 that had BLV provirus detected by both the Single-CoCoMo assay and the Dry Dual-CoCoMo assay had no BLV provirus detected by the Liquid Dual-CoCoMo assay. This sample was positive for the BLV gp51 antibody; however, the BLV PVL measured by the Single-CoCoMo assay in this sample was 13 copies per 10^5^ cells, which may have been because this low PVL was near the limit of detection of the BLV provirus. Moreover, one cow (S40) was negative for gp51 antibodies and positive for BLV provirus. Regarding the BLV provirus detection probability in this cow, all three CoCoMo methods successfully detected the BLV provirus (2/2; 100%). In support of the accuracy of the CoCoMo method, this sample was also detected as positive using the TaqMan-based RC202 qPCR assay targeting the BLV *pol* region [50]. Therefore, these data suggest that blood samples may be taken from cows during the early stages of infection before the antibody levels begin to rise. This is consistent with previous results indicating that BLV was detected in 73.3% (1039/1417) of cattle using the BLV-CoCoMo-qPCR-2 assay, and the provirus was detected in 93 of the 1039 antibody-negative samples [35]. In addition, these data are supported by the fact that the kinetics of the provirus did not precisely correlate with the change in anti-BLV antibody titers in two cattle experimentally infected with BLV [20]. These results indicated that the BLV provirus detection assay using the CoCoMo methods is a necessary diagnostic technique for BLV eradication programs.

In general, the Dual-CoCoMo assays we developed, including the Liquid and Dry Dual-CoCoMo assays, have the following three advantages. First, BLV provirus detection can be performed quickly and inexpensively because of savings in terms of the reagents, laboratory equipment, and working time [51,52,53]. Secondly, the amount of sample used in these assays was only half that of the original BLV-CoCoMo-qPCR-2 assay, allowing for the quantification of valuable and limited samples [53]. Third, human errors such as pipetting can be minimized by simplifying the complicated preparation of PCR reagents [54,55,56]. In addition, the dried reagents in the Dual-CoCoMo assays can be stored at room temperature, thus streamlining their transportation and storage [54,55,56,57]. Therefore, as BLV is a global problem, our Dry Dual-CoCoMo assay, which can be transported anywhere in the world, is expected to become increasingly important for BLV diagnosis and research in the future.

Many researchers have reported the following quantitative PCR methods for BLV diagnosis: TaqMan real-time BLV pol PCR [48,50,58], TaqMan MGB BLV pol real-time PCR [49], SYBR Green real-time BLV pol PCR [59,60], Droplet Digital env and pol PCR [61], and Droplet Digital BLV env PCR [62]. Currently, there is no designated standard protocol or uniform criterion for the quantitative diagnosis of BLV; therefore, it is used according to the purpose of individual studies [63]. Jaworski et al. conducted a validation study for six available BLV qPCR assays to evaluate their inter-laboratory variability. According to their results, the copy number varied greatly among different laboratories, indicating that comparing the quantitative PVLs was difficult. We have also shown in previous studies that our developed BLV-CoCoMo-qPCR-2 assay can detect BLV proviruses with higher sensitivity and reproducibility than other methods and that it can detect various BLV strains of broad geographical origin, including those from Japan, Peru, Bolivia, Chile, and the USA [23,35,36]. Therefore, we expect that our newly developed CoCoMo method will be used as a global protocol for BLV diagnosis in the future and will significantly contribute to the control and eradication of BLV, which is widespread in many countries worldwide.

## Figures and Tables

**Table 1:** BLV proviral load (PVL) as determined by BLV-CoCoMo Dual qPCR using two qPCR master mixes and BLV-CoCoMo-qPCR-2.

Sample No.	Single-CoCoMo ^a^		Dual-CoCoMo ^b^			
			TBIRD ^c^		GeneAce ^d^	
	Mean PVL ^e^	SD ^f^	Mean PVL	SD	Mean PVL	SD
B1	112	18	44	21	51	8
B2	435	38	175	24	318	29
B3	937	85	520	45	894	76
B4	3027	147	2079	224	3155	224
B5	5902	300	6699	465	7730	301
B6	12,046	521	13,950	588	15,175	678
B7	14,883	365	18,699	1369	19,680	329
B8	40,870	1941	44,153	3765	47,304	1369
B9	63,016	2118	61,627	3799	66,705	2516
B10	119,317	2804	104,926	5260	116,959	4202

^a^ Single-CoCoMo: BLV-CoCoMo-qPCR-2 assay. ^b^ Dual-CoCoMo: BLV-CoCoMo Dual qPCR assay. ^c^ TBIRD: THUNDERBIRD Probe qPCR Mix. ^d^ GeneAce: GeneAce Probe qPCR Mix II. ^e^ Mean PVL: the mean BLV proviral load in 10^5^ cells amplified from three samples in three independent experiments. ^f^ SD: Standard deviation.

**Table 2:** Intra- and inter-assay reproducibility measured by BLV-CoCoMo Dual qPCR using two qPCR master mixes and BLV-CoCoMo-qPCR-2.

Method	qPCR Master Mix	Sample No.	Proviral Load (Copies/10^5^ Cells)			Intra-Assay CV ^f^ (%)			Inter-Assay CV ^g^ (%)
			Exp. 1 Mean (SD ^e^)	Exp. 2 Mean (SD)	Exp. 3 Mean (SD)	Exp. 1	Exp. 2	Exp. 3	Exp. 1~3
Single-CoCoMo ^a^	TBIRD ^c^	B1	129 (7)	93 (6)	114 (16)	5.7	6.9	13.6	16.1
		B2	420 (15)	425 (47)	461 (44)	3.6	11.0	9.6	5.1
		B3	910 (16)	944 (160)	957 (41)	1.8	16.9	4.2	2.6
		B4	2997 (164)	3039 (63)	3044 (231)	5.5	2.1	7.6	0.9
		B5	6015 (318)	5764 (219)	5927 (404)	5.3	3.8	6.8	2.2
		B6	12,030 (585)	11,895 (224)	12,213 (786)	4.9	1.9	6.4	1.3
		B7	15,019 (423)	14,929 (412)	14,702 (321)	2.8	2.8	2.2	1.1
		B8	41,771 (1971)	41,638 (959)	39,201 (1997)	4.7	2.3	5.1	3.5
		B9	64,189 (1713)	62,639 (3337)	62,219 (806)	2.7	5.3	1.3	1.6
		B10	119,641 (2699)	121,469 (1137)	116,841 (2562)	2.3	0.9	2.2	2.0
Dual-CoCoMo ^b^	TBIRD	B1	33 (10)	30 (9)	68 (13)	29.3	29.9	19.6	48.4
		B2	182 (19)	189 (13)	155 (30)	10.5	6.8	19.5	10.2
		B3	528 (57)	517 (56)	514 (41)	10.8	10.9	8.0	1.4
		B4	2119 (325)	1923 (113)	2196 (153)	15.4	5.9	7.0	6.8
		B5	6131 (22)	7008 (277)	6958 (242)	0.4	4.0	3.5	7.4
		B6	13,827 (238)	13,381 (251)	14,641 (198)	1.7	1.9	1.4	4.6
		B7	17,967 (1237)	18,099 (902)	20,031 (1072)	6.9	5.0	5.4	6.2
		B8	41,453 (604)	41,897 (259)	49,109 (924)	1.5	0.6	1.9	9.7
		B9	60,183 (1327)	58,469 (1537)	66,229 (1933)	2.2	2.6	2.9	6.6
		B10	101,333 (2817)	102,531 (1014)	110,915 (4468)	2.8	1.0	4.0	5.0
	GeneAce ^d^	B1	57 (10)	47 (4)	49 (7)	18.1	9.1	14.2	10.4
		B2	306 (27)	323 (30)	324 (36)	8.8	9.3	11.1	3.2
		B3	912 (26)	903 (140)	867 (31)	2.8	15.5	3.6	2.7
		B4	3178 (279)	3240 (158)	3048 (263)	8.8	4.9	8.6	3.1
		B5	7768 (170)	7800 (305)	7621 (462)	2.2	3.9	6.1	1.2
		B6	15,025 (338)	15,776 (224)	14,725 (891)	2.3	1.4	6.0	3.6
		B7	19,689 (346)	19,911 (110)	19,441 (366)	1.8	0.6	1.9	1.2
		B8	47,481 (823)	48,245 (1476)	46,185 (1176)	1.7	3.1	2.5	2.2
		B9	69,026 (655)	66,257 (2987)	64,833 (1524)	0.9	4.5	2.3	3.2
		B10	117,881 (3506)	119,300 (5001)	113,695 (2800)	3.0	4.2	2.5	2.5

^a^ Single-CoCoMo: BLV-CoCoMo-qPCR-2 assay. ^b^ Dual-CoCoMo: BLV-CoCoMo Dual qPCR assay. ^c^ TBIRD: THUNDERBIRD Probe qPCR Mix. ^d^ GeneAce: GeneAce Probe qPCR Mix II. ^e^ Mean (SD): the mean BLV proviral load in 10^5^ cells (standard deviation) amplified in triplicate and repeated three times. ^f^ Intra-assay CV: Coefficient of variation between each sample. ^g^ Inter-assay CV: Coefficient of variation between each experiment.

**Table 3:** Comparison of the BLV provirus detection frequency in each qPCR assay.

pBLV-IF 2 ^a^ (Copy Number)	BLV Provirus Detection Frequency (%)					
	Single-CoCoMo ^b^		Liquid Dual-CoCoMo ^c^		Dry Dual-CoCoMo	
100	3/3 ^d^	(100)	3/3	(100)	3/3	(100)
50	3/3	(100)	3/3	(100)	3/3	(100)
25	3/3	(100)	3/3	(100)	3/3	(100)
12.5	3/3	(100)	3/3	(100)	3/3	(100)
6.25	3/3	(100)	3/3	(100)	2/3	(67)
3.125	8/10	(80)	7/10	(70)	7/10	(70)
1.5625	4/10	(40)	3/10	(30)	3/10	(30)
0.78125	3/10	(30)	1/10	(10)	0/10	(0)
0	0/3	(0)	0/3	(0)	0/3	(0)

^a^ BLV infectious full-length molecular clone of BLV (pBLV-IF2) [39]. ^b^ Single-CoCoMo: BLV-CoCoMo-qPCR-2 assay. ^c^ Dual-CoCoMo: BLV-CoCoMo Dual qPCR assay using GeneAce Probe qPCR mix II. ^d^ Number detected per number tested.

**Table 4:** Comparison of the different BLV detection qPCR methods using samples from 82 cattle.

Sample	ELISA ^a^	Proviral Load ^b^ (Copies/10^5^ Cells)			Sample	ELISA	Proviral Load (Copies/10^5^ Cells)		
		Single-CoCoMo	Liquid Dual-CoCoMo	Dry Dual-CoCoMo			Single-CoCoMo	Liquid Dual-CoCoMo	Dry Dual-CoCoMo
BLV-uninfected cattle					BLV-infected cattle without lymphoma				
J2	-	0	0	0	S42	+	8130	12,271	11,065
J5	-	0	0	0	S56	+	8132	12,168	10,102
J9	-	0	0	0	H63	+	9168	8393	12,971
H10	-	0	0	0	H82	+	9342	6379	11,372
J16	-	0	0	0	S24	+	9810	13,238	12,318
J24	-	0	0	0	S54	+	11,242	14,063	13,927
J29	-	0	0	0	H69	+	11,844	10,444	12,204
H28	-	0	0	0	J39k	+	12,183	10,536	11,956
A1	-	0	0	0	J23k	+	12,702	14,566	14,549
A2	-	0	0	0	H57	+	13,435	16,964	17,127
A3	-	0	0	0	C77	+	13,441	11,529	12,966
BLV-infected cattle without lymphoma					J84	+	15,019	19,689	15,142
S23	+	13	0	5	J42	+	17,012	17,014	18,212
S7	+	24	2	4	H4	+	18,874	21,916	21,665
S34	+	26	4	13	H75	+	20,212	22,598	20,631
J8	+	32	3	15	S39	+	21,042	27,235	24,285
S10	+	33	11	34	S43	+	23,270	28,199	26,686
Bs1	+	37	1	9	J41k	+	23,927	21,800	21,335
S40	-	38	3	14	J17k	+	25,710	27,802	24,704
S37	+	88	24	66	S50	+	26,127	34,154	31,848
S14	+	96	35	90	sS1	+	26,131	26,053	23,207
S35	+	110	53	103	S52	+	27,660	37,228	35,544
C78	+	129	57	15	sS3	+	28,132	28,830	25,113
C95	+	276	69	170	C72	+	31,463	27,133	25,000
S11	+	386	274	462	H85	+	33,257	44,667	39,258
H100	+	420	306	207	H103	+	35,373	37,492	36,080
S53	+	567	517	839	H3	+	35,952	37,429	33,172
H65	+	910	912	564	S13	+	37,760	47,798	41,548
S44	+	1657	2062	2092	J37k	+	38,719	39,600	37,791
S36	+	1891	2159	2140	J22k	+	40,527	44,114	42,501
S21	+	2081	2657	2822	H102	+	41,553	46,496	37,861
C60	+	2997	3178	1914	H80	+	41,771	47,481	40,279
S51	+	3116	4172	3982	J21	+	42,014	45,401	41,479
S49	+	4866	5767	5854	sS2	+	43,500	44,218	41,095
S28	+	4956	6022	6314	J18k	+	47,530	48,853	45,261
S55	+	5402	6457	5822	H107	+	47,893	54,363	48,606
S12	+	5565	7740	7038	J7k	+	52,898	51,397	50,210
H106	+	5722	6075	7377	H87	+	64,189	69,026	64,248
H88	+	6015	7768	4893	H51	+	119,641	117,881	120,377
J58	+	6849	7672	7945	BLV-infected cattle with lymphoma				
C94	+	7309	5943	8482	E3	+	42,080	37,035	36,511
H81	+	7882	6493	9152	E57	+	118,919	118,257	131,025

^a^ ELISA testing was performed using an anti-BLV ELISA kit (Nippon Gene Inc.) targeting BLV gp51. +, positive for anti-BLV antibodies; -, negative for anti-BLV antibodies. ^b^ Proviral loads per 10^5^ cells in blood were measured by each qPCR method.

**Table 5:** Comparison of the results from the Liquid Dual-CoCoMo (a) or Dry Dual-CoCoMo assays (b), and Single-CoCoMo assay.

(a)		Single-CoCoMo		Total
		Positive	Negative	
Liquid Dual-CoCoMo	Positive	70	0	70
	Negative	1	11	12
Total		71	11	82
		Sensitivity (%)	Specificity (%)	
		(95% CI)	(95% CI)	
		98.6	100	
		(92.4–100)	(71.5–100)	
(b)		Single-CoCoMo		Total
		Positive	Negative	
Dry Dual-CoCoMo	Positive	71	0	71
	Negative	0	11	11
Total		71	11	82
		Sensitivity (%)	Specificity (%)	
		(95% CI)	(95% CI)	
		100	100	
		(94.9–100)	(71.5–100)	

CI, confidence interval.

## Data Availability

The original contributions presented in the study are included in the article; further inquiries can be directed to the corresponding author.

## Supplementary Materials

The following supporting information can be downloaded at https://www.mdpi.com/article/10.3390/v16071016/s1, Table S1: Primers and probes used for the Dual-CoCoMo assay.
