# Supplementary material for: Development of Dry and Liquid Duplex Reagent Mix-Based Polymerase Chain Reaction Assays as Novel Tools for the Rapid and Easy Quantification of Bovine Leukemia Virus (BLV) Proviral Loads
Source: Viruses. 2024 Jun 25;16(7):1016. doi: 10.3390/v16071016 (PMC11281531; doi:10.3390/v16071016)
Supplement: Supplementary file 1 [file viruses-16-01016-s001.zip › viruses-3044843-supplementary.pdf]

Table S1. Primers and probes used for the Dual-CoCoMo assay

Target gene	Primer/probe	Sequence[a]
BLV LTR	Primer-Fw	5′-AATCCMNMYCYKDAGCTGCTGAYYTCACCT-3′<br>5′-ATCCACACCCTGAGCTGCTGCACCTCACCT-3′<br>Both primers were mixed in 1:10
	Primer-Rv	5′-TTGCCTTACCTGMCSSCTKSCGGATAGCCGA-3′
	Probe	5′-FAM-CTCAGCTCTCGGTCC-NFQ-MGB-3′
BoLA-DRA	Primer-Fw	5′-CCCAGAGTATGAAGCTCCAGCCC-3′
	Primer-Rv	5′-CCCTCGGCGTTCAACGGTGT-3′
	Probe	5′-VIC-TGTGTGCCCTGGGC-NFQ-MGB-3′

[a]The sequences of primers and probes used were previously reported by Takeshima *et al*. [36].
